# Supplementary material for: Are carbon emissions trading and green financial instruments synergistic? -Comprehensive quantitative research based on content analysis
Source: PLoS One. 2024 Mar 7;19(3):e0298601. doi: 10.1371/journal.pone.0298601 (PMC10919601; doi:10.1371/journal.pone.0298601)
Supplement: S1 Data — (ZIP) [file pone.0298601.s001.zip › Data/地级市Z_F工作报告.zip!/╣π╢1⁄2/╜╥╤⌠╩╨/└┤╫╘╣1⁄2╓┌║┼í╛┬φ┐╦╩2╛▌═°í┐_2006.docx]

各位代表：

我代表市人民政府向大会作政府工作报告，请予审议，并请政协各位委员和其他列席人员提出意见。

“十五”时期的工作回顾

“十五”时期，是我市深化改革、加快发展、全面提高的时期，是克难奋进、开拓创新、真抓实干的时期。五年来，在省委省政府和市委的正确领导下，在市人大及其常委会和市政协的监督支持下，市政府坚持以邓小平理论和“三个代表”重要思想为指导，全面贯彻落实科学发展观，狠抓发展第一要务，积极应对各种困难和挑战，采取有效措施，组织强势推进，全市经济社会持续健康协调发展。2005年，生产总值420.4亿元，比上年增长11.2%，五年年均（下同）增长8.7%；地方财政一般预算收入11.1929亿元，比增22.04%，年均增长6.31%。产业结构逐步优化，三次产业比例发展为16.9∶49.3∶33.8。

—— 生产持续稳步发展

认真落实一系列扶持发展的政策措施，促进生产全面发展。工业生产增速较快。纺织服装、五金机械、化工塑料、食品医药和电子信息等支柱产业不断发展壮大。榕泰实业、康美药业、巨轮模具、运通塑料等4家企业成功上市。百事可乐公司在揭东设立生产基地，实现世界500强企业落户我市零的突破。省著名商标从1件增至46件，市知名商标从零增至119件，创立国家名牌产品2个、免检产品4个，省级名牌产品14个；先后荣获“中国五金基地市”、“亚洲玉都”、“中国玉都”称号，普宁市和揭东开发区分别荣获“中国纺织产业基地市”、“中国优秀开发区”称号。2005年，工业总产值806.03亿元，比增15.8%，年均增长11.6%；其中规模以上工业产值301.99亿元，比增19.6%，年均增长16.2%。农业生产平稳发展。制订并推广应用7个省级、26个市级农业地方标准。普宁青梅、揭东竹笋2个省级农业现代化示范区和35个市级以上标准化示范区建设顺利推进，6大农业支柱产业基本形成，12大农业商品基地建设规模不断扩大。18家省、市级农业龙头企业带动作用进一步增强，27家年销售额1000万元以上农产品加工企业发展势头良好。普宁荣获中国“青梅之乡”、“蕉柑之乡”和“青榄之乡”称号，揭东、惠来分别荣获“竹笋之乡”、“荔枝之乡”称号。2005年，农林牧渔业总产值111.42亿元，比增5.1%，年均增长4.4%。

—— 内外经贸取得成效

采取“政府搭台、企业唱戏”等形式，大力开拓国内外市场，搞活搞旺内外经贸。对外贸易发展较快。五年新增外贸进出口经营权企业234家，累计370家。2005年，外贸出口11.32亿美元，比增19.4%，年均增长13.4%。会展经济有效拓展。积极参加“广交会”、“珠洽会”、“中博会”等各类商品展销会，成功举办中国（揭阳）国际玉器节、普宁（国际）衬衣节暨纺织服装交易会、中国（普宁）中药材交易会和揭西生态旅游文化节等节会，揭货市场占有率不断提高。市场建设得到加强。世界500强正大集团的零售企业易初莲花在市区落户。粤东鞋材城、市水果批发和普宁中药材、纺织服装等市场逐步配套完善，市区金属材料、粮食市场和普宁粤东烟草物流配送中心等正在抓紧建设。消费需求稳中趋旺。旅游、汽车、通讯、餐饮、商住等消费热点增长较快，物业、社区、中介等服务业稳步发展。2005年，社会消费品零售总额143.35亿元，比增13.7%，年均增长11.7%。

—— 基础建设有效推进

调整优化投资结构，加快交通、能源、水利等基础设施和市政建设。全社会固定资产投资五年累计436.63亿元，年均增长10.1%。交通建设进展顺利。普惠、揭普、梅揭等高速公路建成通车，新改建地方公路1705公里；潮汕民用机场、汕揭和潮揭高速公路以及南河大桥扩建等工程正在抓紧推进，厦深铁路揭阳段规划正在论证。能源建设取得成效。惠来电厂、石碑山风电场建设步伐加快，累计已分别投入资金22.5亿元、5.71亿元。第一、二期城乡电网改造工程全面完成，新（扩）建220千伏变电站 2座、110千伏变电站9座、风能电厂1座，供电网络进一步完善。水利建设扎实开展。五年共投入资金19.74亿元，修建、加固江海堤围95.18公里，完成横江、上坑、汤坑等中型水库和地都、东陇等江海堤围的除险加固工程，抗灾能力不断增强。城乡建设步伐加快。引资30亿元建设市区城市设防综合工程及配套市政设施加快推进，榕东大桥、梅东大桥、环市北河大桥、岐山汽车站、黄岐山森林公园等建成投入使用，市垃圾处理场首期工程建设和临江北路、黄岐山大道改造基本完成，马牙路改造、临江南路综合工程、莲花大道南段、市体育馆、揭阳楼、榕江大型音乐喷泉、市区污水处理厂等一批市政设施正在抓紧建设，市容市貌有所改观。各县（市、区）每年筹资 1 亿元以上投入城区市政公共设施建设任务得到落实，全市镇级规划编制工作基本完成，16个中心镇新一轮总体规划修编工作进展顺利。

—— 体制改革不断深化

积极稳妥推进各项改革，初步建立起适应社会主义市场经济的运行机制。国有企业改革稳步推进。市棉纺厂、电焊机厂等企业改制遗留问题得到妥善解决，揭阳糖厂、氮肥厂等国企改制步伐加快。国有资产监管机制不断完善。财政管理体制逐步健全。市、县、镇三级财政管理体制进一步理顺，部门预算、政府采购、国库集中支付等改革有效实施，公共财政体制框架初步形成。地方金融改革稳妥推进。财政资金追收和金融资产清收力度不断加大，财政金融风险逐步化解。农信社改革试点工作稳步开展，地方金融秩序保持稳定。社会保障体系基本建立。社保扩面征缴取得重大进展，2005年组织企业社保费收入2.37亿元，比增115.17%。企业养老保险实现市级统筹，医疗、失业、工伤保险制度得到有效实施。国企下岗职工基本生活保障和城乡居民最低生活保障制度得到落实。农村等各项改革深入开展。农村土地资本化改革稳步推进，第二轮土地承包工作基本完成。农村税费改革成效明显，农民人年均负担减少68.1元。撤并规模偏小的乡镇12个，降低行政管理成本。投融资和粮食流通、食品药品监管、质量技术监督等各项改革顺利进行，市场经济体制进一步完善。

—— 社会事业协调进步

坚持以人为本，积极推动各项社会事业与经济建设同步发展。教育事业发展加快。教育基础设施建设力度加大，2005年投入资金5.6亿元，新扩建中小学99所，五年累计455所；改造老区山区破旧小学160所，五年累计341所；揭阳学院、市体育运动学校等相继建成，揭阳一中、市技校新校区等正在加紧建设。师生素质得到提高，2005年增配教师2377人，其中本科学历540人；初中升普通高中3.1856万人，每万人口在校高中生123.7人，比“九五”期末增加68.6人。科技工作取得成效。揭阳高新技术产业开发区、市仙梅民科园先后经省批准设立。五年共实施国家和省各类科技计划159项；新增国家、省高新技术企业27家，省级民营科技企业64家；成立省级工程技术研发中心4个、博士后工作站2个。与华工大、华农大合作进展顺利。公共卫生得到加强。抗击非典和禽流感疫情取得重大胜利。公共卫生体系逐步健全，应急指挥系统不断完善。疾病防控工作进一步落实，卫生监督力度加大，新型农村合作医疗农业人口覆盖率达39.1%。其他事业稳步发展。国防动员、兵员征集、海边防建设、民兵预备役和双拥优抚安置工作进一步加强。文化、体育、人防、物价、统计、气象、地震、外事侨务、民族宗教、文史档案、广播电视、新闻出版等各项事业取得新进步。

—— 各项管理力度加大

切实加强各项管理，努力维护社会和谐稳定。市场秩序逐步规范。偷税逃税、制假售假、走私贩私等违法犯罪活动受到严厉打击，非法传销、无照生产经营等问题得到有效整治，建设领域政府项目拖欠工程款问题基本解决，旅游、土地、食品、药品等市场秩序进一步规范。交通运输、危险化学品等专项整治深入开展，安全生产形势基本稳定。生态管理不断加强。人口与计生工作扭转被动落后局面，稳步走上依法管理轨道。殡改成果继续巩固，火化率保持100%。环保目标任期责任制进一步落实，榕江、练江、枫江水污染得到遏制，“一控双达标”工作取得成效。土地、矿产、森林、海洋等资源保护力度加大，耕地总量实现动态平衡，生态公益林体系、绿色通道及三年灭荒工程有效实施，森林覆盖率51.5%。揭西被列为“国家生态示范区建设县”。社会治安得到整治。以打黑除恶、打击“两抢一盗”、“六合彩”赌博等为重点的严打整治斗争取得成效，“禁毒03工程”扎实推进，普宁摘除“毒品重灾区”帽子。“法轮功”邪教组织破坏活动受到有力打击。基层调解、信访工作不断加强，群体性、突发性事件得到妥善处置解决，社会局面基本保持稳定。

—— 人民生活继续改善

积极推进十项民心工程，认真解决关系民生民计的热难点问题，不断提高人民生活质量和水平。2005年，市区城镇居民人均可支配收入9192元，农民人均纯收入4126元，年均分别增长7.4%、3.3%。城乡统筹就业扎实推进。五年累计新增就业岗位7.5万个，国有企业下岗职工实现再就业3.5万人，转移农村劳动力就业42万人，城镇登记失业率控制在3%以内。社会救助工作顺利实施。五年累计发放低保金1.34亿元，15.01万人次享受低保救济待遇；拨出救灾救济款5076万元，救济灾民167万人次。扶贫开发力度不断加大。五年共投入资金3.81亿元，实施支农扶贫项目1520个，44个贫困村基本脱贫达标。群众“四难”问题得到缓解。饮水工程扎实推进，解决了120多万农村群众饮水难问题；农村安居工程有效实施，共落实危房改造资金9700万元；司法服务和法律援助力度逐步加大；农村困难群众子女教育扶助政策得到落实。

—— 民主法制进程加快

坚持科学行政、依法行政、民主行政，加强民主法制建设，塑造良好政府形象。民主建设继续推进。认真执行人大及其常委会的决议、决定，定期向人大常委会报告工作、向政协通报情况，自觉接受监督。加强与工会、共青团和妇联等群团的联系，认真听取各民主党派、工商联和无党派人士的意见建议。五年共办理人大代表建议267件、政协委员提案388件。基层民主继续扩大，村（居）民自治制度逐步完善。法制建设扎实开展。行政许可法全面实施，行政执法责任制和过错责任追究制进一步落实。城市管理相对集中处罚权试点工作顺利铺开，行政执法行为和行政复议工作更加规范。“四五”普法任务按期完成，全民法制观念不断增强。自身建设得到加强。政府机构改革顺利完成，市级政府机构、行政编制分别精简27.3%、35.5%。清理和取消审批项目591项，减幅达61%。审计监督成效明显，受到国家表彰。行政监察力度加大，廉政建设和反腐败斗争深入开展，市政府向社会公开作出并认真履行五项廉政承诺。机关作风建设不断加强，行政效率和服务水平进一步提高。

各位代表，“十五”时期，我市在比较困难的情况下，保持经济社会稳步发展，取得了较好成绩。这是省委省政府和市委正确领导的结果，是各级人大及其常委会和人民政协监督支持的结果，是全市人民团结拼搏、共同努力的结果，是中央和省驻揭单位、驻揭人民解放军、武警官兵、各民主党派、人民团体、港澳台同胞、海外侨胞、海内外乡亲和友好人士关心支持的结果。在此，我代表市人民政府表示衷心的感谢并致以崇高的敬意！

回顾过去五年，我们也清醒地看到，我市经济社会仍存在不少困难和问题。突出表现在：产业结构不够合理，经济增长质量偏低，整体经济实力不强；企业普遍“小、弱、散”，自主创新能力较弱，工业发展水平不高；投资软硬环境有待改善，招商引资效果仍不理想，投资拉动相当乏力；市区辐射作用不强，县域经济发展滞后，城镇化进程缓慢；村镇经济仍较薄弱，农业基础建设投入不足，农民收入增长不快；市场秩序有待深入整顿和规范，安全生产仍然存在薄弱环节，环境污染亟待治理；不稳定因素依然存在，治安形势仍较严峻，维护稳定压力较大；部分干部艰苦创业意识不浓，精神状态不够振奋，机关作风有待继续改进。对此，我们要采取更加有力措施，在加快发展中认真解决。

“十一五”时期的奋斗目标和主要任务

“十一五”时期，是我市实现跨越发展的重要时期。市政府工作的指导思想是：以邓小平理论和“三个代表”重要思想为指导，以科学发展观统领经济社会发展全局，认真贯彻落实中央十六届五中全会和省委九届七次、八次全会以及市委三届四次全会精神，积极实施工业强市、文化大市、商贸旺市、三农优先、绿色揭阳 “五大战略”，突出组织工业发展、城镇建设、基础教育“三个突破”，增强自主创新能力，转变经济增长方式，强势加快经济发展，协调发展社会事业，推进民主法制进程，保持社会和谐安定，努力实现经济社会科学、健康、跨越发展。

“十一五”时期，我市经济社会发展的主要奋斗目标：生产总值年均增长12%，单位生产总值能耗比“十五”期末降低13%以上；工业总产值年均增长15%，全社会固定资产投资年均增长20%，社会消费品零售总额年均增长15%，地方财政一般预算收入年均增长19%。力争到2010年，经济总量比2000年翻一番以上，三次产业结构调整为11.5∶54.9∶33.6。

完成上述任务，实现奋斗目标，必须进一步解放思想，继往开来，做到“六个更加注重”：在经济增长上，更加注重改善运行质量和效益，力促经济发展向集约型、循环型转变；在体制改革上，更加注重体制和机制创新，力促关键领域改革攻坚取得新突破；在区域发展上，更加注重协调推进，力促尽快形成地区优势互补、相互促进、统筹发展的格局；在文化建设上，更加注重以人为本，力促市民综合素质和社会文明程度明显提高；在维护稳定上，更加注重解决群众热点难点问题，力促社会保持和谐安定；在依法行政上，更加注重改进作风和提高工作水平，力促政府调节经济、管理社会和公共事务能力显著增强。为此，要切实做到“六个加快”：

一、加快产业结构调整，全面提升经济素质

以调整优化经济结构为主线，以增强自主创新能力为核心，切实提高经济增长质量和效益。壮大优化第二产业。鼓励运用先进适用技术改造提升传统产业，积极发展能源工业、化工产业以及电子信息等高新技术产业，推动产业集群优化升级，不断做大做强工业经济。大力发展第三产业。调整优化进出口结构，转变外贸增长方式，加快发展对外贸易。积极参加各类经贸洽谈会，认真办好地方特色产品节会，努力开拓国内市场。加强市场体系规划建设管理，大力发展现代服务业，更有效扩大消费需求。调整提高第一产业。切实保护耕地，严格控制基本农田保护区，稳定和提高粮食生产。巩固发展农业支柱产业，加快农业商品基地和特色水产养殖基地建设。推进农业标准化生产，发展效益农业、特色农业和生态农业，提高农业综合效益。培植壮大农业龙头企业，加快农产品市场建设，健全农业社会化服务体系，提高农业产业化水平。

二、加快基础设施建设，大力改善发展环境

加大投资建设力度，加快基础设施和市政建设，着力改善投资环境。多方筹措建设资金。深化投融资体制改革，进一步简化审批手续和程序，放开投资领域，积极利用民资、外资和侨资，争取国债资金、上级专项补助和银信资金支持，确保建设资金落实配套。全力推进重点建设。以惠来电厂、潮汕民用机场、市区防洪工程为龙头，加快实施能源、交通、水利、环保、文化“五大工程”，建立健全与我市经济社会发展水平相适应、适度超前的基础设施体系。以引资30亿元建设市区城市设防综合工程及配套市政设施为突破口，全力推进市区和县城建设，完善城市功能，提升城市品位。强化工程质量监管。严格实施投资项目绩效综合考评制度，推行工程项目公开招投标，从制度和源头上防止工程建设的腐败现象。加强建设项目质量管理，确保工程质量。

三、加快深化改革开放，建立良好运行机制

加大改革攻坚力度，扩大区域合作与交流，形成改革开放的新格局。推进体制创新。深化国有企业改革，完善现代企业制度。健全国有资产监管体制，加强财务监督和管理。深化财政管理体制改革，完善公共财政体系。推进地方金融改革，规范中小金融机构发展。健全现代市场体系，大力发展产权、资本、劳动力、技术等要素市场。扩大对外开放。积极实施互利共赢的开放战略，主动参与国际竞争与合作，在更大范围、更宽领域、更高层次上扩大对外开放。创新招商机制和方式，提高吸收外资的质量和水平，优化引资和投资结构，推动产业升级。充分发挥侨乡优势，加强对外友好往来，促进对外合作与交流。加强区域协作。推进闽粤赣十三市区域经济协作，加强同“泛珠三角”、“大珠三角”区域的联系与沟通。认真实施与华南理工大学、华南农业大学合作协议，推动产学研合作。

四、加快壮大县域经济，促进城乡协调发展

按照打造“四大产业带”的要求，根据各地基础条件和发展水平的实际，因地制宜，分类指导，加快形成分工合理、重点突出、比较优势得以发挥的县域经济格局。扶持发展民营经济。认真贯彻落实加快民营经济发展的优惠措施，大力发展特色产业，培育一批专、精、特的中小企业群，形成集群产业和块状经济，推动县域经济加快发展。切实推进城镇建设。抓紧完成中心镇总体规划修编工作，积极推进小城镇规划建设。加强中心镇基础设施、公共服务设施建设，逐步完善城镇功能配套，促进城镇第二、三产业发展，增强辐射和带动农村的能力。加强新型农村建设。积极推广玉湖先进经验，加强基层政权建设。全面推进农村综合配套改革，建立农民增收减负长效机制。加强农村规划建设，强化村容村貌综合整治，努力改善农村生产生活环境，建设社会主义新农村。

五、加快建设文化大市，推动社会文明进步

积极发展文化事业，努力提高市民文明素质。加强精神文明建设。把握正确舆论导向，大力弘扬艰苦奋斗、改革创新、锐意进取的时代精神。深入开展“爱国、守法、诚信、知礼”现代公民教育活动，推进公民道德建设。广泛开展科普宣传，革除陈规陋习，促进移风易俗，形成科学、健康、文明的生活方式。大力发展科教事业。建立健全以企业为主体的科技创新体系，加强知识产权保护，大力推动产学研联合，加快科技成果转化。全面落实“以县为主”的基础教育管理体制，巩固和提高“普九”成果，大力普及高中教育，积极推进高等教育、成人教育，鼓励发展民办教育、远程教育，提高教育质量和办学水平。努力繁荣社会文化。深化文化体制改革，推进文化产业化，打造文化精品和文化品牌，大力发展文化经济。继续弘扬潮汕优秀历史文化，加强历史文化名城保护、开发，争创全国优秀旅游城市。强化文化市场管理，规范文化市场秩序，健全文化市场体系。统筹发展其他事业。积极发展卫生、体育、人防、民政、气象、地震、外事侨务、民族宗教、文史档案、广播电视、新闻出版等各项事业。

六、加快推进协调管理，努力构建和谐揭阳

加大统筹协调、强化管理力度，为加快发展营造稳定和谐的社会环境。加强利益协调。建立健全社会利益协调机制和收入分配调节机制。高度重视民生民计，关心帮扶弱势群体，着力解决群众切身利益问题，努力化解各种社会矛盾。注重生态建设。强化人口、资源、环境综合管理，积极发展循环经济，切实保护生态环境，加快建设绿色揭阳，促进人与自然和谐相处。维护社会稳定。整顿和规范市场经济秩序，严厉打击各种经济违法行为。加强安全生产监督管理，防止重特大安全事故的发生，确保人民群众生命财产安全。进一步做好信访和社会矛盾排查调处工作。深入开展社会治安综合治理，依法严厉打击“黄赌毒”、黑恶势力等违法犯罪活动，大力推进治安防控体系建设。切实转变职能。深化行政管理体制改革，推进政府管理创新和制度创新，把政府职能切实转变到经济调节、市场监管、社会管理和公共服务上来，提高依法行政水平，增强保障公共安全和处置突发事件的能力，保持社会和谐稳定。

各位代表：展望“十一五”发展蓝图，我们充满信心。只要全市人民树立艰苦奋斗的创业精神，鼓足坚韧不拔的工作干劲，奋力拼搏，真抓实干，揭阳将会以崭新的形象崛起在南粤大地！

2006 年的工作安排

2006年，是实施“十一五”规划的开局之年，做好今年的工作，为“十一五”规划顺利实施开好头、起好步，至关重要。今年我市经济社会发展主要预期目标：生产总值增长12%，单位生产总值能耗下降 2.3%，工业总产值增长16%，农林牧渔业总产值增长4%，全社会固定资产投资增长25%，外贸出口增长15%，实际利用外资增长10%，社会消费品零售总额增长15%，地方财政一般预算收入增长19.94%，城镇登记失业率控制在3.5%以内，人口自然增长率控制在7.5‰以下。

实现上述目标，必须着力抓好10方面工作：

一、发展壮大工业经济，着力优化产业结构

大力培育产业集群。继续支持和规范传统优势产业发展，做大做强五金制品、塑料制品、纺织服装、食品医药等传统支柱产业，积极发展电力能源、机械制造、石油化工等新兴主导产业，研究制定中医药发展规划和配套政策，努力推进中医药现代化。狠抓技术改造和创新，重点抓好巨轮模具、榕泰实业等40个技改项目的实施工作。加快技术研发、信息服务、质量检测、物流配送等公共服务平台建设，推动产业优化升级。

扶持发展龙头企业。继续实行领导干部挂钩扶持重点企业制度，认真落实扶持工业发展的优惠措施，在用电补贴、贷款贴息、专项资金等方面扶持优势企业，特别要大力扶持纳税额5000万元以上尤其是上亿元企业，推动一批企业做大做强。继续实施“三个新增100”，努力壮大工业规模。引导企业加快公司制、股份制改造，大力鼓励和支持有条件的龙头企业上市，提高融资能力和资本运营水平。

做大做强品牌产品。发挥现有品牌的经济效应和示范作用，积极鼓励、引导有条件的企业申报品牌和商标，培育壮大一批拥有自主知识产权、核心竞争力强的品牌企业，力争新增1件驰名商标、1个免检产品和2个名牌产品。

强化园区建设管理。创新园区经营理念，引入市场机制，转变办园模式，将园区建设管理由政府行为转变为企业行为，推动工业园区良性滚动发展。继续实施“668”计划，重点抓好揭东开发区、惠来沿海试验区、榕城仙梅民科园、普宁高柏工业基地、揭西棉湖和东山磐东工业集中区等工业发展载体的建设管理。积极创造条件，争取尽快启动惠来石化产业园区和市级工业园。

二、强势推进城区发展，着力构筑区域核心

科学规划开发。进一步修编完善市区总体规划，加快编制和完善专项控制性详规，抓紧编制“十一五”近期建设规划，认真抓好莲花大道至试验区的城市新区规划，搞好五千年中华历史文化景观长廊规划设计。严格控制、科学开发市区181平方公里内未开发土地和两河四岸建设线之外纵深150米土地，切实加强县（市、区）城区土地控制和规划管理。加快旧城和城中村改造。做好“水”字文章，建设富有水乡特色的生态城市。

加快市政建设。全力推进引资30亿元建设市区城市设防综合工程及配套市政设施，加快临江南路、莲花大道南段、淡浦路南段等市政道路建设，完善道路交通标志、信号灯和“电子眼”等配套设施。加快揭阳楼、榕江大型音乐喷泉等市政设施建设，进一步完善城市功能，提高城市品位。继续落实各县（市、区）筹集1亿元以上资金投入城区市政公共设施建设任务，进一步改变城区面貌，打造区域发展次中心。

搞活城市经营。学习借鉴先进地区的经验做法，引入市场机制，探索推行科学的城市公共资源经营管理模式，有效盘活、整合城市公共资源。加大土地招标、拍卖和挂牌出让力度，合理配置土地资源，实现土地增值。科学引导房地产开发，大力营造优良的人居环境。对市政道路开挖、管线占用、市政设施冠名权、户外广告设置、城市公交线路经营权等资源实行有偿使用和出让经营，走以城建城、以城养城、滚动发展的路子。

强化综合管理。确立“大城管”理念，进一步理顺城市管理体制，合理划分确定市与区、部门与部门之间的事权，推动工作重心下移，强化责任落实，调动各级各部门参与城市管理积极性和主动性。推进市政公用事业改革，实行“以费养事”，逐步推行园林绿化管护、环卫保洁市场化管理。加强市区和县城综合管理，深入开展交通、卫生、绿化等专项整治，综合治理“脏、乱、差、噪”，努力改善市容市貌，树立良好的城市形象。

三、狠抓重点项目建设，着力夯实发展基础

推进能源建设。加快惠来电厂建设，确保明年初首台机组投产发电，力争惠来石碑山风电场今年建成投产。开工建设500千伏惠来电厂至榕江送电线路以及500千伏榕江和220千伏紫峰、铁山等重点输变电工程，完成500千伏长沙至揭阳、220千伏棉湖至紫峰送电线路以及220千伏揭阳、棉湖变电站扩建和华湖输变电工程建设。继续推进城乡电网改造，不断完善电网结构。

推进交通建设。加快潮汕民用机场建设前期工作，力争今年开工建设。抓紧汕揭和潮揭高速公路揭阳段、国道206线揭丰段、省道236线池揭段改造、南河大桥扩建工程建设，协助做好厦深铁路规划和筹建工作。尽快完善揭阳港规划，加紧推进靖海码头建设。加快地方公路改建步伐，进一步完善交通网络。

推进水利建设。加快榕城围南北堤、渔湖围南北堤等水利设施建设，确保按期建成。积极推进惠来石榴潭水库和赤岑水闸、揭东磐岭围、普宁流沙联围新河堤加固、揭西金凤联围和县城防洪工程等项目建设，提高城乡水利防灾减灾能力。

四、加大市场拓展力度，着力搞旺内外经贸

大力发展对外贸易。切实转变外贸增长方式，优化出口商品结构，积极扩大外贸出口，提高出口的质量和效益。继续巩固现有市场，大力开拓新兴市场。认真办好泰国揭货博览会。加强与海关、检验检疫、税务、外汇管理等部门的协调和沟通，加快电子口岸建设，营造“大通关”环境。

千方百计招商引资。健全招商引资工作机制，强化招商工作责任考核，调动各级招商积极性。创新招商方式，扩大招商领域，优化投资环境，以更加优惠的条件引进大公司、大企业特别是跨国公司、世界500强企业到我市落户。认真做好集聚民心民资民力工作，广泛动员揭籍在外经济大户回乡投资置业。

努力开拓国内市场。组织企业参加第三届泛珠洽会、第三届中博会等各类经贸洽谈会，扩大揭货市场占有率。办好中国（揭阳）国际玉器节、普宁（国际）衬衣节暨纺织服装交易会、揭阳（国际）五金不锈钢制品博览会等节会，促进会展经济发展。认真做好市区商业网点规划，抓紧筹建沃尔玛购物广场和大润发购物中心，加快市区汽贸一条街、金属材料、粮食市场和五金制品交易中心建设，做大做强普宁中药材、纺织服装、烟草物流配送等专业市场。认真实施“万村千乡”市场工程，进一步完善农村商品流通网络。大力发展旅游、社区和中介服务，培育形成消费热点。

五、积极发展农村经济，着力建设新型农村

提高农业综合效益。稳定和提高粮食生产能力，大力发展效益农业、特色农业和生态农业。加大新品种、新技术、新机具推广力度，巩固提高6大农业支柱产业、12大农业商品基地发展水平，切实加强市农科所蕉柑良种无病毒苗木繁育中心、普宁果树中心、惠来特色水产养殖基地、揭西蕉柑苗圃基地和有机茶生产示范基地建设。发展壮大18家省、市级农业龙头企业，积极培育新的龙头企业，建立健全农民专业合作经济组织和农业服务体系，提高农业产业化水平。

加强农村基础建设。切实搞好社会主义新农村规划，积极推进中心镇建设，加快农村道路、饮水、电网、通讯等基础设施建设步伐。认真抓好社会主义新农村建设试点，为全面铺开建设提供借鉴。加强村容村貌整治，重点推进改水、改路、改房、改厕、改灶“五改”工作，努力改变农村环境“脏乱差”现象，建设干净、整洁、优美的和谐文明新农村。

强化农村各项管理。认真做好农村第二轮土地承包后续管理工作。加快征地制度改革，健全对被征地农民的合理补偿机制和安置机制。进一步加强村账镇管和财务监督。探索化解镇村集体债务的有效办法，稳妥化解镇村债务。完善基层民主管理制度，积极推行村务公开。建立健全农村干部退休退职基金制度，调动基层干部工作积极性。

努力增加农民收入。坚持“多予少取放活”的方针，进一步深化以农村税费改革为主要内容的综合配套改革，加大扶农惠农力度，发展壮大镇村集体经济。加强农民就业培训和技工教育，重点抓好“百万农村青年技能培训工程”和创业培训工程，不断提高农村劳动力素质，引导农村富余劳动力向非农产业和城镇有序转移，提高农民收入水平。

六、突出抓好财税社保，着力增强财力保障

依法强化财税管理。通过调整体制、兑现扶持政策，加大对骨干企业、纳税大户的扶持力度，培育壮大地方税源。坚持依法治税，加强税收征管，规范非税收入管理，严厉打击偷税、逃税、骗税、抗税等违法犯罪行为，防止税源流失，确保应收尽收。调整优化支出结构，集中财力保障人员工资、社会稳定等重点领域支出。加强财税监管和审计监督，进一步规范财经秩序。

狠抓社保扩面征缴。加大社保费征缴力度，强化社保费征缴责任考核。进一步健全社保费征收机制，规范征缴行为，完善后续管理工作，做实个人帐户。以非公有制企业为重点，加强宣传教育，扩大社会保险覆盖面。严格基金支出管理，确保社保待遇按时足额发放。

深化财政金融改革。推进财政管理体制改革，健全财税激励机制，调动基层组织收入积极性。加大财政资金和金融资产追收处置力度，依法、稳妥做好城信社停业整顿和退出市场工作，防范和化解地方财政金融风险。加快农信社改革步伐，支持发展金融保险产业，促进地方金融机构规范稳健发展。

七、高度关注民生民计，着力改善人民生活

努力增加就业岗位。大力拓宽就业渠道，鼓励自主创业。建立健全市场导向的就业机制，加强职业技能培训和技工教育，提高劳动者素质和就业能力，促进就业再就业。加快公共就业服务体系建设，规范发展劳动力市场，完善劳动力市场信息网络，有效引导劳动力合理流动。

强化社会保障工作。积极推进企业退休人员社会化管理，探索建立农村养老保险、医疗保险制度，逐步完善以养老、失业、医疗、工伤和生育保险为主要内容的社会保障体系。认真落实城乡最低生活保障制度，确保应保尽保。

加大扶贫助困力度。切实做好救灾救济工作，保障困难群众的基本生活。推进农村饮水工程建设，缓解群众饮水难状况，提高农村群众饮水安全水平。建立健全社会救助体系，努力解决困难群众看病难、入学难、住房难、打官司难等问题。

八、加快发展社会事业，着力提升文明程度

优先发展教育事业。继续落实资金筹措、校舍建设和提高师生素质三大任务，强化基础教育责任考核。努力推进农村免费义务教育，从今年秋季起全面免除农村户籍学生义务教育阶段杂费。加快普通高中教育发展，积极创建示范性高中。大力发展职业教育和技工教育，继续办好揭阳职业技术学院。加大教育基础设施建设投入，加快揭阳一中新校区、揭阳侨中新校区、揭阳学院附中扩建工程建设步伐。做好教师资格认定工作，加强教师培养、培训和引进，提高教师队伍整体素质。

切实加强科技工作。认真落实省委省政府关于提高自主创新能力、提升产业竞争力的决定，制订出台相关配套措施。积极培植高新技术企业和民营科技企业，大力支持企业设立研发机构，打造自主创新基地。力争新增5家高新技术企业、1个省级工程技术研发中心。依法保护知识产权，做好科普工作，提高全民科技意识。加强人才培养引进，营造有利于优秀人才脱颖而出的创业环境。

积极繁荣社会文化。搞好历史文化名城保护和开发，切实加强文物保护。扶持发展玉器工艺等地方特色优秀文化，大力发展文化产业。积极推进市体育馆、档案馆、榕城文化广场、东湖公园等文化设施建设，认真做好市文化中心（揭阳文化广场、市广电中心、图书馆、艺术学校、青少年活动中心、博物馆、群艺馆）前期工作。加快县、镇公共文化设施和农村广播站建设步伐，丰富城乡群众文化生活。

大力推进其他事业。加强卫生应急机制建设，健全公共卫生应急体系。强化疾病控制和卫生监督，重点抓好非典、禽流感、艾滋病等重大传染病的防控工作。推进城镇医疗卫生体制改革，规范药品集中招标采购、医疗服务项目和收费标准。加快市人民医院住院大楼等医疗卫生设施建设，抓紧规划建设市卫校新校区。加强农村卫生服务网络建设，完善新型农村合作医疗制度，力争覆盖率达到40%以上。广泛开展群众性体育运动，发展体育产业。加强国防安全教育，做好双拥工作。发展老龄、妇幼和残疾人事业。做好外事、侨务、台务、宗教、统计、民政、人防、气象、地震、档案、方志、文史等工作。

九、切实强化各项管理，着力维护社会稳定

强化生态管理。认真抓好人口与计生工作，严格控制人口增长，确保完成全年工作任务。加强殡葬设施建设，继续巩固殡改成果。严格落实环保目标任期责任制和追究制，加大环保执法力度，依法取缔、关闭污染严重的企业，强化工业污染控制和城乡环境综合整治，着力抓好榕江、练江、龙江等流域水资源保护。加快市区和县城垃圾处理场、污水处理厂等环保工程建设，逐步完善城乡治污保洁配套设施。加强土地、矿产、森林、海洋等资源保护与管理，推进资源节约和综合利用。

强化市场管理。继续整顿和规范市场经济秩序，严厉打击 制假售假、走私贩私、非法传销等违法犯罪行为。加强食品药品监管，大力实施食品放心工程，抓好重特大事件的预防预警和应急处理工作，保障人民群众身体健康。加强安全生产监督管理，用铁的手腕遏制重特大安全生产事故的发生。

强化社会管理。认真做好人民调解和信访工作，依法依规处置好人民群众来信来访事件。加强流动人口和出租屋管理，消除社会管理“盲点”，积极创建“平安村”和“平安社区”。加大社会治安综合治理投入，加快全天候治安视频监控网络、中心法庭和司法所建设。保持高压态势，依法严厉打击“两抢一盗”、绑架勒索、吸毒贩毒、赌博等违法犯罪活动，切实解决治安突出问题，增强群众安全感，保障人民安居乐业。

十、加强民主法制建设，着力提高行政效能

推进民主建设。坚持向人大及其常委会报告工作、向政协通报情况的制度，自觉接受监督。及时办理人大代表议案、建议和政协委员提案。认真听取各民主党派、工商联、无党派人士和工青妇等群团组织的意见建议，推行专家咨询、社会公示和听证等制度，提高政府决策科学化、民主化、法制化的水平。

促进依法行政。认真贯彻落实《全面推进依法行政实施纲要》，严格按照法定权限和程序行使权力，规范行政行为。深化行政审批制度改革，推行政务公开，坚持依法决策、依法办事、依法处理问题。实施“五五”普法规划，增强公务员队伍的法制观念。

改进机关作风。树立艰苦奋斗的创业精神，大兴求真务实之风，积极为基层和群众多办好事实事。切实精简会议、文件，狠抓各项决策和工作部署的检查落实。加强电子政网、行政服务中心建设，提高行政效率和办事水平。减少各类评比活动，建立健全科学的政府绩效评估体系和经济社会发展综合评估体系。深入开展廉政建设和反腐败斗争，认真预防和严厉惩治腐败犯罪，坚决纠正损害群众利益的不正之风。加强教育培训，建设政治坚定、业务精通、清正廉洁、作风优良的公务员队伍。

各位代表：承继“十五”业绩，开创“十一五”辉煌，是时代赋予我们的光荣使命。让我们在市委坚强有力的领导下，同心同德，振奋精神，开拓进取，扎实工作，为完成“十一五”规划各项任务，建设富裕、文明、和谐的新揭阳而努力奋斗！
